# The Usability of Homelab, a Digital Self-service at a Dutch General Practice, for Diagnostic Tests: Pilot Study With a Questionnaire

**DOI:** 10.2196/42151

**Published:** 2023-01-26

**Authors:** Kyma Schnoor, Anke Versluis, Niels H Chavannes, Esther P W A Talboom-Kamp

**Affiliations:** 1 Public Health and Primary Care Leiden University Medical Center Leiden Netherlands; 2 National eHealth Living Lab Leiden Netherlands; 3 Unilabs Geneva Switzerland

**Keywords:** eHealth, diagnostic testing, general practitioner, general practice, GP, referral, online testing, diagnostic, laboratory test, usability, digital health, health care service, service delivery

## Abstract

**Background:**

eHealth potentially can make health care more accessible and efficient and help reduce the workload in primary health care. Homelab is an eHealth tool implemented in a general practice environment, and it offers relatively simple laboratory diagnostic tests without the referral of the general practitioner. After logging in this eHealth tool, patients select and order a diagnostic test based on their symptoms. The test results are presented online to the general practitioner and the patient.

**Objective:**

This study aims to evaluate the use, usability, and user characteristics of Homelab. Further, it aims to evaluate whether Homelab replaces an appointment with the general practitioner.

**Methods:**

Homelab has been implemented since May 2021 as a pilot in a Dutch general practice. The number of requests and the ordered diagnostic packages are monitored. After using Homelab, patients are invited to complete a short questionnaire. The questionnaire contains demographic questions and assesses usability using the System Usability Scale (10 items). In addition, questions about requesting an appointment with the general practitioner without Homelab are included. All data were anonymous.

**Results:**

The questionnaire was filled by 74 individual patients. The mean age of the patients was 40.33 (SD 12.11) years, and half of them were females (39/74, 53%). The majority of the patients were highly educated (56/74, 76%) and employed (53/74, 72%). Approximately 81% (60/74) of the patients reported that they would use Homelab again in the future and 66% (49/74) reported that they would have gone to the general practitioner if they had not used Homelab. The usability of Homelab was perceived higher by the younger age group (mean 73.96, SD 14.74) than by the older age group (mean 61.59, SD 14.37). In total, 106 test packages were ordered over 1 year, and the most requested diagnostic package was “Am I still healthy? I want to do my annual health checkup.” Homelab was used the most during the months of the COVID-19 lockdown.

**Conclusions:**

The use of Homelab, a digital self-service for ordering diagnostic tests, was monitored in this study, and its usability was perceived as above average. Our findings showed that patients are willing to use Homelab in the future and they would use it most of the time as a replacement for regular consultations. Homelab offers opportunities for more accessible and efficient health care for both the patient and the general practitioner.

## Introduction

The number of patients with chronic diseases is high and is increasing worldwide [1,2], thereby leading to a high workload for health care professionals, especially in primary care, as many patients require complex care [3]. General practitioners (GPs) have a positive attitude toward innovations like eHealth [4-6]. eHealth can be defined as “health services and information delivered or enhanced through the internet and related technologies” [7]. eHealth can potentially lower the workload of GPs [4,5]. For example, in the Netherlands, a noncommercial website was developed by GPs for citizens to obtain reliable health information [8], and a significant decrease in the consultations was noted after the website’s launch compared to the total consultations before the launch [8,9]. Apps that support lifestyle change or the self-management of chronic diseases (eg, promoting physical activity, healthy diet, weight management) can also benefit GPs, as these apps can take over part of the GPs’ coaching [10-12]. Consequently, GPs may have more time for other health care activities.

The COVID-19 pandemic accelerated the development and use of technology in health care with more web-based consultations and home monitoring [13,14]. One study showed that using technology in health care increased accessibility because it was easy for patients to use web-based consultations [13]. eHealth gives patients more control of their health, and it has the potential to increase self-management [15]. A way to use eHealth effectively is to integrate eHealth into regular care—the so-called hybrid care or blended care; in this way, eHealth can be used more frequently, which may positively impact health care outcomes [16].

One area where eHealth can be used is laboratory diagnostic testing with direct access to diagnostic tests and result services. With such services, patients can order a diagnostic test online, for example, for COVID-19, perform the test at home or a facility, and view the result online. A recent review [17] showed that most of the included web-based diagnostic services (which were operated independently by health care professionals) were positively evaluated and found very acceptable by patients, but most of the services focused on sexually transmitted infections, and direct access to diagnostic services for other diseases was rare.

Our study describes a new diagnostic-related eHealth initiative called Homelab, which is a direct web-based access service implemented in the environment of the general practice. Patients can use Homelab to order diagnostic tests online without going to the GP for a diagnostic test referral. After ordering a test on Homelab, the patient’s GP needs to authorize the ordered test; this way, GPs can monitor what is being ordered. Authorizing the ordered tests ensures that the tests are reimbursed health care. A consultation is scheduled when a diagnostic test result is abnormal or a disease or a condition is present. Both the patient and the GP can view the test result online.

To our knowledge, this is the first web-based diagnostic service completely integrated into the web-based environment of the GP, and no research has been performed into the type of users and the frequency of use of Homelab. Although services are available where patients can order diagnostic tests themselves without a GP [17], a service where this is integrated in the GP environment is new. Homelab has several advantages for the patient. First, patients do not need a GP consultation for a diagnostic test referral, and the patient can thus quickly order a diagnostic test online. Second, Homelab can help a patient prepare for the GP consultation, as the diagnostic test result can be viewed online beforehand. This way, Homelab may help to empower the patient and increase consultation efficiency. Further, it may save time for the GP because the GP does not have to perform consultations for relatively simple diagnostic test referrals; consequently, GPs may have more time for more complex cases. Another critical aspect of the Homelab service is reimbursed health care. In a previous review [17], web-based diagnostic services were not part of reimbursed health care, and the patient had to pay the costs. Costs, however, were a barrier to using such services [17].

Homelab was implemented as a 1-year pilot in 2021 in a general practice in the Netherlands, making it possible to research a direct access diagnostic service in the environment of the GP. This pilot study aims to identify who uses Homelab, how and how often Homelab is used, and how patients perceive its usability. Furthermore, the aim of this study was to identify whether using Homelab potentially replaces an appointment with the GP.

## Methods

### Study Design and Population

A quantitative pilot study was conducted between April 21, 2021 and April 4, 2022. User characteristics and user experiences were collected through questionnaires, and data on how often Homelab was used (eg, what and how many tests were ordered) were extracted from Homelab. The data were not linked to each other due to privacy legislation. Homelab was implemented as a pilot at the Westerdokters General Practice in Amsterdam; this practice is known for its innovation and digitization. The study population consisted of registered patients at the Westerdokters General Practice who chose to use Homelab. There were no exclusion criteria for participation. All the patients of the Westerdokters General Practice could use Homelab.

### The Service: Homelab

**Figure 1:**
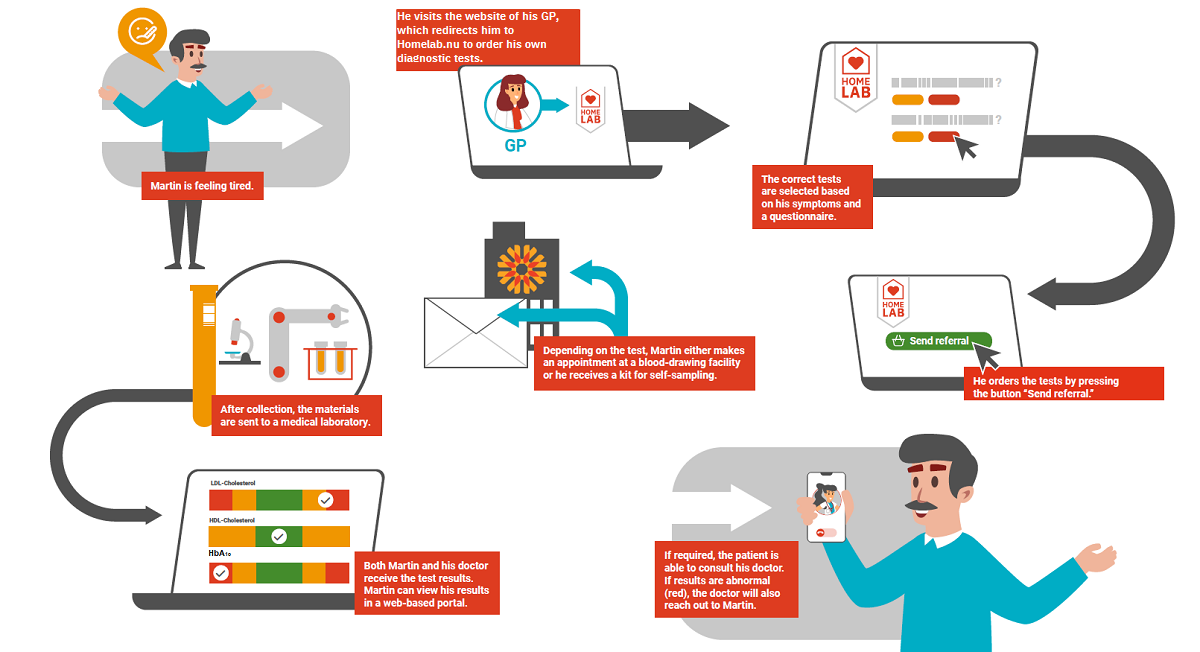
Patient journey with Homelab. GP: general practitioner; HbA1_c_: hemoglobin A1_c_; HDL: high-density lipoprotein; LDL: low-density lipoprotein.

Homelab is a Dutch digital self-service that offers patients direct access to diagnostic tests. This service is accessible from the website of the general practice. The test packages ordered on Homelab are frequently requested and are standard diagnostic tests, for example, diagnostic tests for anemia or fatigue. Unilabs developed Homelab in cocreation with Dutch GPs. In Figure 1, the patient journey is presented. Unilabs is an international diagnostic provider, which offers laboratory, imaging, and pathology specialties in 16 countries [18].

First, patients visit the GP’s website and log in via a 2-factor authentication. Second, patients can select a health problem (see Textbox 1; eg, I feel tired; what is wrong?). Third, patients complete follow-up questions related to the selected health problem (eg, Have you been tired for several weeks or months, and is this affecting your life?). The questions are based on medical guidelines (triage). Fourth, after the digital triage, a combination of specific diagnostic tests, further referred to as test package(s), is suggested to the patient. It could also be that an explanation is given without a diagnostic test referral. Fifth, the patient can order the recommended test package(s), and the GP can authorize or cancel the requested test package(s). Depending on what kind of materials (eg, feces, urine, blood) are required for testing, the patient can make an appointment for blood sampling at the general practice or hand in their urine sample or feces at the general practice. After the analysis of the materials (eg, feces, urine, blood) in a professional medical laboratory, results are presented in a secure tailor-made web-based portal and available for both the patient and GP [19,20]. An electronic consultation can be initiated by the patient or the GP when the results are concerning or if the patient has questions.

**The list of health problems that can be selected on Homelab (translated from Dutch to English).**

- I feel tired; what is wrong?
- Am I still healthy? I want to do my annual health checkup.
- Am I allergic?
- What is my blood type?
- Why do I often have pain in my stomach?
- Why can I not lose weight?
- Do I have anemia?
- Do I have an elevated prostate-specific antigen? (only available for men)
- Why do I have hair loss?
- Is my body system free of any traces of drugs?

### Outcome Measures

#### Questionnaire Data: Demographic and Clinical Characteristics

The following demographic characteristics of Homelab users were assessed: year of birth, gender, education level, and employment status. Low education was defined as primary school or prevocational secondary education; intermediate education included upper secondary education and vocational education; and high education was defined as graduated from universities of Applied Sciences, research universities, and doctoral degree programs. For employment status, there were different categories: student, which was defined as a pupil (secondary school and student); employed (defined as having a full-time or part-time job, or being an entrepreneur); voluntary work, retired, or unemployed, which was defined as being unemployed or unable to work (eg, due to sickness or incapacity for work); or other. Finally, patients were asked whether they had a chronic disease. Answer options were “yes, asthma/chronic obstructive pulmonary disease;” “yes, cardiovascular disease;” “yes, diabetes;” or “no, none of the above.”

#### Questionnaire Data: Homelab Use

To gain insight into how Homelab was used, 3 questions were asked. The first question was on using Homelab as a replacement for consultation. To investigate whether patients would have gone to the GP if they did not have access to Homelab for a diagnostic test, we asked the following question: If you did not order a diagnostic test via Homelab, would you have gone to the GP? The answer options were yes, no, and I don’t know.

The second question determined whether patients would like to have the possibility of ordering diagnostic tests independent of the GP in the future. The following question was asked: Would you like to have the possibility of ordering diagnostic tests online independent of a GP in the future? Answer options were yes, no, and I don’t know.

The third question was on the costs of using Homelab. In this pilot study, Homelab could be used for free by patients. Generally, in the Netherlands, the costs of diagnostic tests ordered at the general practice are covered by the health care insurance or by the patient when the patient’s medical costs in that year are below €385 (US $418) (ie, the standard amount of obligatory, deductible excess in 2021). To identify whether patients would order the test if they had to pay for it themselves, the following question was asked: I would also order this test when it would come at the expense of the deductible of my health insurance. The answer items were rated on a 5-point Likert scale ranging from strongly disagree to strongly agree.

#### Questionnaire Data: System Usability Scale

The System Usability Scale-10 items (SUS-10) is a valid and robust questionnaire to determine whether a system is user-friendly and can be used for an app or website [21]. The questionnaire consisted of 10 items (eg, I think that I would like to use this app frequently). Each item was rated on a 5-point Likert scale ranging from strongly disagree to strongly agree. The negatively formulated items were reversed scored. The sum score of all the items was multiplied by 2.5 to obtain the total SUS score. The SUS total score ranges from 0 to 100, where a higher score means that the app is more user-friendly [21]. A score above 68 is considered usability above average [22].

### Ordered Test Packages

Data on the number of ordered test packages and the type of ordered test packages were collected. This information was downloaded via a content management system function of Homelab. These anonymized data were not linked to the questionnaire data. Therefore, data were not traceable to an individual participant, and the data were anonymous.

### Procedure

On the Westerdokters Practice website, a link to Homelab was provided. Homelab was explained to patients in the general newsletter of Westerdokters twice. After the patients ordered a diagnostic test, they had the possibility of completing the questionnaire. At the start of the questionnaire, there was a short introduction about the study aim, expectations from participants, and why the study was performed. Patients were not obliged to fill in the questionnaire. From the beginning of the pilot study until January 2022, Homelab users could complete the questionnaire multiple times (ie, every time they ordered diagnostic test package(s) on Homelab). In January 2022, this was corrected, and patients could only complete the questionnaire once. All data were downloaded via a content management system of Homelab.

### Ethical Considerations

Approval by an ethics committee was not needed for this study because no intervention or trial has occurred in the sense that the research participants were subjected to actions or had modes of behavior imposed on them. Obtaining informed consent and ethical approval was unnecessary because the questionnaire data were anonymously collected. The data on the frequency of Homelab use were anonymous.

### Statistical Analyses

Descriptive statistics (eg, mean [SD], total sample, percentages, frequencies) were used to summarize all the demographic and clinical characteristics, number and type of orders of test package(s), and data on SUS-10. Moreover, the data were split for age (≤40 years and >40 years) and gender, and descriptive statistics were used to give insight into these different groups. The analyses were performed using SPSS version 25 (IBM Corp) [23].

As described above, there was a fault in the programming, and patients could complete the questionnaire multiple times. If patients ordered multiple packages on Homelab (at the same time), the patient would be presented with the questionnaire after every ordered test package. In the final data set, however, we wanted patients to be only represented once. Therefore, we looked at the demographic characteristics of successively incoming data points. When the demographic data of the next row(s) were identical, we looked at the SUS data of these rows. If there was variation in the SUS data in the first row but not in the consecutive row(s) (ie, all items scored with a 3), we assumed that the consecutive row(s) were of the same patient and were therefore removed from the final data set.

## Results

### Descriptive Statistics

In total, 79 questionnaires were completed. Data from 5 questionnaires were removed because these data were from individuals (n=3) who completed the questionnaire multiple times, resulting in a total of 74 patients with valid questionnaires. Table 1 presents the demographic and clinical characteristics of the patients and their use of Homelab data. The mean age of the patients was 40.33 (SD 12.11; range 23-73) years; half of them were females (39/74, 53%), and the majority were employed (53/74, 72%) and highly educated (56/74, 76%). Furthermore, most did not have asthma, chronic obstructive pulmonary disease, diabetes, or cardiovascular diseases (69/74, 93%).

**Table 1:** Demographics and clinical characteristics of Homelab users (N=74).

Characteristic		Total	Age ≤40 years	Age >40 years	Male	Female
Age, mean (SD)		40.33 (12.1)	31.95 (3.8)	50.76 (10.7)	41.44 (12.1)	38.64 (11.3)
**Gender, n (%)**						
	Male	34 (46)	17 (42)	17 (52)	N/A^a^	N/A
	Female	39 (53)	24 (59)	15 (46)	N/A	N/A
	Unknown	1 (1)	0	1 (3)	N/A	N/A
**Education, n (%)**						
	Low	5 (7)	0	5 (15)	3 (9)	1 (3)
	Intermediate	13 (18)	7 (17)	6 (18)	9 (27)	4 (10)
	High	56 (76)	22 (67)	22 (67)	22 (65)	34 (87)
**Employment status, n (%)**						
	Student	5 (7)	2 (5)	3 (9)	5 (15)	0
	Employed	53 (72)	34 (83)	19 (58)	25 (74)	28 (72)
	Unemployed	6 (8)	3 (7)	3 (9)	1 (3)	5 (13)
	Voluntary work	0	0	0	0	0
	Retired	7 (10)	0	7 (21)	3 (9)	3 (8)
	Other	3 (4)	2 (5)	1 (3)	0	3 (8)
**Chronic diseases, n (%)**						
	Asthma/chronic obstructive pulmonary disease	4 (5)	0	4 (12)	3 (9)	1 (3)
	Cardiovascular diseases	1 (1)	1 (2.4)	0	0	1 (3)
	Diabetes	0	0	0	0	0
	No	69 (93)	40 (98)	29 (88)	31 (91)	37 (95)
**Replacement for consultation^b^, n (%)**						
	Yes	49 (66)	24 (59)	25 (76)	24 (71)	25 (64)
	No	9 (12)	8 (20)	1 (3)	4 (12)	5 (13)
	I don’t know	16 (22)	9 (22)	7 (21)	6 (18)	9 (23)
**Future use of Homelab^c^, n (%)**						
	Yes	60 (81)	36 (88)	24 (73)	26 (76)	34 (87)
	No	1 (1)	1 (2)	0	0	1 (3)
	I don’t know	13 (8)	4 (10)	9 (27)	8 (24)	4 (10)
**Willing to use if it came at the deductible expense of my health insurance^d^, n (%)**						
	Totally agree	19 (26)	8 (20)	11 (33)	9 (27)	10 (26)
	Agree	16 (22)	10 (24)	6 (18)	7 (21)	9 (23)
	Neutral	19 (26)	14 (34)	5 (15)	7 (21)	11 (28)
	Disagree	14 (18)	6 (15)	8 (24)	8 (24)	6 (15)
	Totally disagree	6 (8)	3 (7)	3 (9)	3 (9)	3 (8)
System Usability Scale (10 items), mean (SD)		68.45 (15.7)	73.96 (14.7)	61.59 (14.4)	67.94 (15.3)	69.30 (16.3)

^a^N/A: not applicable.

^b^This variable was based on the question, “If you did not order a diagnostic test via Homelab, would you have gone to the general practitioner?”

^c^This variable was based on the question, “Would you like to have the possibility of ordering diagnostic tests online independent of a general practitioner in the future?”

^d^This variable was based on the statement of “I would also order this test when this would come at the expense of the deductible of my health insurance.”

### Use of Homelab

Of the total patient population, 66% (49/74) reported that they would have gone to the GP if they had not used Homelab, while 22% (16/74) reported that they did not know if they would have gone. The percentage of patients in the younger age group (24/41, 59%) who would have gone to the GP was lower than that of patients in the older age group (25/33, 76%). Moreover, the percentage of male patients (24/34, 71%) who would have gone to the GP was higher than that of female patients (25/39, 64%). Of the total patient population, 81% (60/74) wanted to use Homelab again in the future without going to the GP, while 8% (13/74) did not know if they wanted to use it again. The percentage of patients in the younger age group (36/41, 88%) who would use Homelab again in the future was higher than that of patients in the older age group (24/33, 73%). In addition, the percentage of female patients (34/39, 87%) who would use Homelab again was higher than that of male patients (26/34, 76%). Almost half of the patients (35/74, 47%) (totally) agreed with the statement, “I would also order this test when this would come at the expense of the deductible of my health insurance,” and about a quarter (20/74, 27%) (totally) disagreed with the statement. The percentage of patients in the younger age group (18/41, 44%) who (totally) agreed with this statement was slightly lower than that in the older age group (17/33, 52%). For both females (19/39, 49%) and males (16/34, 47%), the percentage that (totally) agreed was almost equal.

### Usability of Homelab

The mean score on the SUS-10 was 68.45 (SD 15.74; range 40-100), which can be considered above average usability. The average SUS score in the younger age group (mean 73.96, SD 14.74) was higher than that in the older age group (mean 61.59, SD 14.37). There did not appear to be gender differences (females, mean 69.30, SD 16.29; males, mean 67.94, SD 15.29).

### Ordered Test Packages

The number of unique users of Homelab was 76. The total number of diagnostic test packages that were ordered was 106. In the beginning, Homelab was not used very often (n=3); in May, a few days after the release, Homelab was not used at all. In June, July, August, September, October, November, and December of 2021, Homelab was used 14, 8, 5, 5, 9, 4, and 6 times, respectively. In January and February of 2022, Homelab was used the most (22 times in both months). Table 2 gives an overview of the types of diagnostic test packages that were ordered and how often they were ordered. The most ordered test package was “Am I still healthy? I want to do my annual health checkup” (51/106, 48.1%). The second and third most ordered test packages were “I feel tired; what is wrong?” (24/106, 22.6%) and “Am I allergic?” (9/106, 8.5%), respectively. One test package was not ordered (Is my body system free of any traces of drugs?).

**Table 2:** Overview of the diagnostic packages and frequency of ordering the packages (N=106).

Package name	Values, n (%)
Am I still healthy? I want to do my annual health checkup.	51 (48.1)
I feel tired; what’s wrong?	24 (22.6)
Am I allergic?	9 (8.5)
What’s my blood type?	7 (6.6)
Do I have anemia?	4 (3.8)
Do I have an elevated prostate-specific antigen? (only available for men)	4 (3.8)
Why do I often have pain in my stomach?	3 (2.8)
Why can I not lose weight?	2 (1.9)
Why do I have hair loss?	2 (1.9)
Is my body system free of any traces of drugs?	0

## Discussion

### Principal Findings

Our study identified the characteristics of Homelab users, how and how often the diagnostic service was used, and its usability. The main users of Homelab were highly educated and employed. The age range of the users was broad, but the mean age of the studied population was comparable to that of the Dutch population in 2022 (40.3 years old vs 42.3 years old, respectively) [24]. Patients used Homelab in two-thirds of the cases instead of going to a GP; 81% (60/74) of the patients were willing to use it in the future and half of the patients would also order diagnostic test packages when it came at the expense of the deductible part of their health insurance. Thus, the usability of Homelab was perceived as above average.

The usability of Homelab was perceived higher by younger patients than by older patients, which is in line with that reported in other research on eHealth services [20,25]. Research shows that younger patients are more digitally competent than older patients and are more used to a web-based world [26], potentially making it easier for them to use an app such as Homelab and thereby explaining the higher usability score among younger patients. Older patients may have scored the usability lower because they may have specific wishes and needs (eg, having face-to-face contact with their GP); older patients may have more physical problems or chronic diseases where a normal consultation with the GP might be more preferred [27]. The wishes and needs of older patients could result in lower scores on the items of the usability questionnaire, such as willing to use Homelab in the future. Indeed, most patients who visit the GP are older; in the Netherlands, two-thirds of the consultations are performed with patients older than 40 years [28,29]. Although the usability of Homelab perceived by older patients was lower than that perceived by younger patients, the usability was still perceived as average. Future research should be performed to investigate how Homelab could be beneficial and seamlessly meet the needs of users of this specific older age group to improve its usability [30,31].

This pilot study was also set up to identify if patients would use Homelab excessively because they could order the diagnostic test packages themselves. However, the number of ordered tests was not very high in the pilot period, and it seemed that there was no excessive use. Although it is too early to draw conclusions, Homelab seems to show potential in replacing consultations with the GP without excessive and unnecessary use (based on the number of ordered tests found in this pilot in combination with the answers to the question, “If you did not order a diagnostic test via Homelab, would you have gone to the GP?”).

This was the first study performed on a web-based service for patients, allowing them to order diagnostic test packages in the digital environment of the GP without needing a consultation. Other studies have evaluated services with direct access to laboratory diagnostic testing and results, but those services were without a health care professional [17]. In Homelab, the GP is involved to ensure that patients receive proper care. Still, for the GP, Homelab requires a minimum of time investment. Our results suggest that patients were willing to use Homelab in the future, and they used this service instead of going to the GP, which suggested that they are willing to replace the physical consultation with Homelab. Publications on other digital apps also showed a decrease in consultations when eHealth was used [8,9].

A previous study [32] that researched the usability of another kind of direct access to a diagnostic service was comparable to that of Homelab. However, it [32] was not performed in the GP environment. That study [32] found that the service to order diagnostic tests for sexual transmitted infections online was easy to use (an element of the SUS), which was in line with the results of this study. Our study is the first to describe a web-based service for diagnostic tests where patients can order diagnostic tests themselves in the general practice environment. However, an important part of this service is a tailor-made results portal where patients can view their results online. The results portal was not investigated in this study, but previous studies have examined the benefits of presenting results online [19,33]. Research shows that more than one-third of the studied population was positive about accessing their diagnostic test results online [33], and the usability of the web-based results portal was rated positively [19]. More research is needed to address the efficiency and usability of Homelab.

### Limitations and Strengths of This Study

Our study has some limitations. First, Homelab was piloted during the COVID-19 pandemic, which means that there were restrictions in daily life, and a large part of primary health care was shifted to web-based care [13,14]. Thus, it could be that patients were more open to using Homelab in the COVID-19 period, as web-based health care was the norm. If patients were more open to eHealth in that period, this could have led to more positive reactions to Homelab. Especially in the lockdown period in the winter of 2021 in the Netherlands, Homelab was used more than that in the other months. However, the shift to web-based health care possibly remains because the benefits of using eHealth are more well-known now, and patients have a more positive attitude toward eHealth now than before the COVID-19 period [34,35]. Second, data were unavailable on whether patients really used the diagnostic test package that they ordered. For more insight into patients’ follow-ups, the entire patient journey should be analyzed in future research. Third, the general practice where Homelab was piloted was a relatively digital practice; they have a website where patients can make appointments online and have remote consultations (eg, phone calls, chats, video calls) [36]. Patients of this general practice were perhaps more used to eHealth than patients at other less digital general practices, which could influence the perceived usability of Homelab.

A strength of our study was that this is the first pilot study in a real-world setting with a new web-based diagnostic service. This usability study can help in making this service user-friendly and help in receiving the best experience for the user. Points of improvement derived from this study can be used to revise the service [37]. Another strength is that Homelab was developed in cocreation with GPs. Cocreation in eHealth interventions is an important precondition for good adoption of eHealth [11]. Homelab was piloted and developed for general practices in the Netherlands. However, a service like Homelab can also be implemented in other European countries with comparable primary health care systems where the GP is the first gatekeeper—in particular, Nordic countries are relatively advanced in adopting eHealth [11].

### Conclusions

This pilot study describes Homelab, a digital self-service, wherein patients can order diagnostic tests online in the environment of the GP. This eHealth tool was used by a broad age group but not used excessively. Patients were willing to use Homelab in the future, and they used it most of the time as a replacement for regular consultation. The usability of Homelab was perceived as above average and as better in a younger population. More research should be performed to increase the usability of Homelab, obtain more insights into end user’s needs, and examine if Homelab can lead to more efficient and accessible health care for both patients and GPs.

## Abbreviations

GP: general practitioner
SUS: System Usability Scale

## Data Availability

The data sets generated and analyzed during this study are available from the corresponding author on reasonable request.
